# Connected Bradford:  a Whole System Data Linkage Accelerator

**DOI:** 10.12688/wellcomeopenres.17526.1

**Published:** 2022-01-27

**Authors:** Kuldeep Sohal, Dan Mason, John Birkinshaw, Jane West, Rosemary R.C. McEachan, Mai Elshehaly, Duncan Cooper, Rob Shore, Michael McCooe, Tom Lawton, Mark Mon-Williams, Trevor Sheldon, Chris Bates, Megan Wood, John Wright

**Affiliations:** 1 Bradford Institute for Health Research, Bradford Hospitals National Health Service Trust, Bradford, BD9 6RJ, UK; 2 Department of Computer Science, University of Bradford, Bradford, BD7 1DP, UK; 3 Public Health, Bradford Metropolitan District Council, Bradford, BD1 1HX, UK; 4 School of Psychology, University of Leeds, Leeds, LS2 9JT, UK; 5 Institute of Population Health Sciences, Queen Mary University of London, London, E1 4NS, UK; 6 The Phoenix Partnership, Leeds, LS18 5PX, UK

**Keywords:** Routine population data; Local Government; population health; prevention

## Abstract

The richness of linked population data provides exciting opportunities to understand local health needs, identify and predict those in most need of support and evaluate health interventions. There has been extensive investment to unlock the potential of clinical data for health research in the UK. However, most of the determinants of our health are social, economic, education, environmental, housing, food systems and are influenced by local authorities.

The Connected Bradford Whole System Data Linkage Accelerator was set up to link health, education, social care, environmental and other local government data to drive learning health systems, prevention and population health management. Data spanning a period of over forty years has been linked for 800,000 individuals using the pseudonymised NHS number and other data variables. This prospective data collection captures near real time activity.

This paper describes the dataset and our Connected Bradford Whole System Data Accelerator Framework that covers public engagement; practitioner and policy integration; legal and ethical approvals; information governance; technicalities of data linkage; data curation and guardianship; data validity and visualisation.

## Introduction

Routine electronic information about individuals is recorded in large quantities by healthcare professionals across different healthcare settings to identify, investigate, diagnose and treat patients. This information is an important and rich resource that enables healthcare professionals to support patient care, however this information is currently under-utilised and is not always linked for direct care purposes by the clinical systems across the different care settings resulting in a disconnect between healthcare organisations, fragmentation of care and incomplete pathways of care.

Opportunities to link individuals routinely recorded data across healthcare settings including primary care, secondary care, community care, urgent and emergency care and social services could be delivered by a unique reference number (NHS number) that is recorded by healthcare systems.

Using the NHS number, fragmented individual patient contacts within different healthcare settings can be linked within a population health management framework ^1^. The framework offers insights from patient interactions at the GP practice, hospital and community settings. Over the last ten years there has been extensive investment to establish data linkage across health care organisations with the goal of understanding patient pathways and harnessing linked routine data to drive health service improvement and research ^2^.

However, it is accepted that the majority of ill-health is caused by wider determinants of health that lie outside the health service ^3^. The data and intelligence that describes these factors is typically held by non-health care organisations such as local authorities, schools, housing associations, criminal justice, and environmental agencies. Linking non-health data with health service data at a local level and in ways that ensure a live information system usable by decision makers in real time, would transform our ability to understand the upstream influences on health, to design and test interventions to prevent ill-health, and to influence local decision making and shape policy ^4, 5^.

A number of successfully linked data models operating across Bradford provided an ideal environment to use this experience to build a new collaborative solution, integrating research into practice using evidence-based interventions based on epidemiological results. One particularly successful model is the Born in Bradford birth cohort study ^6^. Between 2007-11 the Born in Bradford cohort obtained informed consent for 30,000 participants for linkage of health and education records. While the detailed research and biological data that the cohort collected has led to exciting scientific findings, the most useful intelligence for policy and practice has come from this linked routine data. For example, we were able to demonstrate the associations between air pollution and child health ^7^ and use this evidence to design an ambitious clear air zone for the city. Evidence linking green space and mental health has been used to obtain investment to redesign parks. Linkage between birth data and school attainment ^8^ has led to policy changes in school admissions for children who were premature. Linkage between schools Early Years Foundation Profile and autism diagnoses has been used to completely redesign child autism support ^9^. 

Bradford has built on the Born in Bradford experience to establish a new Connected Bradford Whole System Data Linkage Accelerator for a much wider population that is representative of the entire Bradford population. Connected Bradford (cBradford) covers 800,000 citizens, five NHS Trusts, 86 general practitioners and 200 schools, and links pseudonymised health, education, social care, environmental and local government data. After extensive community consultation and citizen juries, the team have worked closely with NHS and local authority agencies to develop safe and secure data linkage and established a trusted research environment as part of the regional Yorkshire Health Care Record Exemplar (YHCR) ^10^.

## Setting

Bradford is a post-industrial city in the North of England with high levels of deprivation and poor health, and a multi-ethnic population including a large Pakistani community and growing communities of East European and Roma people. Bradford is governed locally by Bradford Metropolitan District Council (BMDC) which is the 4 ^th^ largest metropolitan council in England. BMDC serves a population of 534,300 and covers an approximate area of 141 square miles ^11^. The NHS Bradford District and Craven Clinical Commissioning Group (CCG) came into operation on 1 April 2020 and supports a population of almost 600,000. The CCG commissions hospital services, urgent and emergency care services and supports the Bradford and Craven population with their mental health and wellbeing from Bradford Hospitals NHS Foundation Trust (BTHFT), Airedale NHS Foundation Trust, Bradford District Care Trust, 86 General Practices and other community care organisations. 

Connected Bradford explored existing linked datasets across these providers which were truly characteristic of the population of 600,000 and identified early on that this did not exist. 

Limitations with existing linked datasets including the Born in Bradford study did not capture all age groups within Bradford’s population. Other linked datasets were either anonymised and had no flexibility to link additional datasets including datasets from the wider determinants of health or enable analysts and researchers to explore for secondary use analysis or for research purposes. 

These limitations provided the catalyst to develop the Connected Bradford Whole System Data Linkage Accelerator. 

## Ethical approval

Ethical approval for a research database was sought to provide reassurances to data providers and develop research studies by making use of the linked dataset that covers both the entire Bradford population and the wider Yorkshire region. The Bradford Institute for Health Research applied to the East Midlands – Derby Research Ethics Committee due to the committee’s existing experience with research databases and ethical approval as a research database to Connected Bradford was granted on 31 August 2017 (IRAS ref:227117 and REC ref:17/EM/0254). A further amendment was submitted to the East Midlands – Derby Research Ethics Committee to include a) add additional datasets to the database b) allow GPs to opt out of the wider use of the data for all pathway projects identified by the programme c) allow GPs to opt out of the use of Apollo to extract the pseudo data from the GP practice and d) extend the availability of the database to external researchers. Support from the East Midlands – Derby Research Ethics Committee was granted on 20 March 2019.

However, to support the programme’s vision to link healthcare data with education data, we identified that there would only be a limited number of individuals that would have both their healthcare and education data recorded by the respective organisations. Education records are in existence for those that are born from 1991 to date where individuals have attended schools in the UK and reference a Unique Pupil Number (UPN) which is not made available or recorded routinely by healthcare organisations. Similarly the NHS number is not routinely available to education departments and thereby data linkage using a unique reference number across healthcare and education is not supported. This necessitated the need to identify additional legal bases to use personal non-unique identifiers and the need to develop a new research database for this linked data.

An application for a new research database was submitted to the Yorkshire & Humber Bradford Leeds Research Ethics Committee and Confidentiality Advisory Group (CAG) for s251 approval for approval under Regulation 5 of the Health Service (Control of Patient Information) Regulations 2002 to process confidential patient information without consent. The new application referenced a new research database Connected Bradford Health and Education Research Database (HERD) which identified a specific cohort of over 300,000 individuals that reside in the Bradford region who are registered at one of the 86 General Practices and were born from the year of 1991. The Yorkshire & Humber Bradford Leeds Research Ethics Committee favourable approval supported the application on 3 August 2018 and CAG favourable opinion was granted on 3 September 2018 (IRAS ref: 239924, CAG ref: 18/CAG/0091 and REC ref: 18/YH/0200). A further amendment to the HERD Database was submitted to the respective committees to flow personal identifiers to the Department for Education (DfE) to enable healthcare data to be linked with the National Pupil Database (NPD) and the DfE social care data as well as link Geospatial data. Yorkshire & Humber Bradford Leeds Research Ethics Committee approval was received 25 July 2019. The CAG favourable opinion was granted on 16 December 2019 and 30 July 2020 respectively whilst the DfE formally approved the NPD application on 26 May 2020. More recently, to enable the Connected Bradford research database to be updated on an ongoing basis for the existing cohort of 315,693 individuals and include additional children that start school in Bradford at the beginning of the academic year in September, favourable opinion was granted for this amendment by the CAG committee on 16 April 2021.

### Data acquisition

Information Governance (IG) is a key challenge in accessing data, sharing data, hosting data, using data and developing a linked dataset. Unfortunately, there is very little publicly available guidance that sets out the information governance framework to establish a dataset that can be used be for secondary use analysis and research purposes. Guidance was sought from an independent IG consultant who supported Connected Bradford’s commitment to ensure that data from healthcare, local government and other partners is always shared securely and lawfully. With this support, the programme developed a Data Protection Impact Assessment and data sharing agreements that outlined scope, the data linkage pseudonymisation process, mitigations to identified risks and concerns from engagement activities with data providers and the public, legal bases and information security. Further advice was obtained from the Information Commissioner’s Office, with subsequent buy-in from the Local Medical Committee and the NHS Bradford District and Craven Clinical Commissioning Group, to secure further reassurances for the regional clinical workforce before agreements and information leaflets were distributed to data providers. It then took approximately six months to receive all the signed data sharing agreements from all 86 General Practices across Bradford, the three Trusts serving the Bradford district; BTHFT, Bradford District Care Trust and Airedale NHS Foundation Trust and Bradford Metropolitan District Council. Following this, further agreements have been collated from other data providers in Bradford and across the Yorkshire region to expand and scale the Whole Systems Data Accelerator data linkage programme.

### Pseudonymisation and linkage

Identifiable information is removed at source by the data providers so that personal information is not available to the study. The NHS number is encrypted at source to create a unique but non-identifying linking key. This is obtained using a secure one-way pseudonymisation process to derive an invariant pseudonym from the NHS number to enable data to be linked across multiple organisations at Connected Bradford ^12^. To ensure that the programme would not have any mechanism by which an individual could be intentionally or unintentionally re-identified, an agreement and process was developed with a trusted organisation to hold the encryption key on behalf of the data providers. The encryption key is only shared with named Caldicott Guardians or designated officers in the data providers that have signed agreements with the programme. De-identified data is then joined to other datasets that used the same process using the non-identifying linking key, which is subsequently removed from production databases.

**Figure 1.**
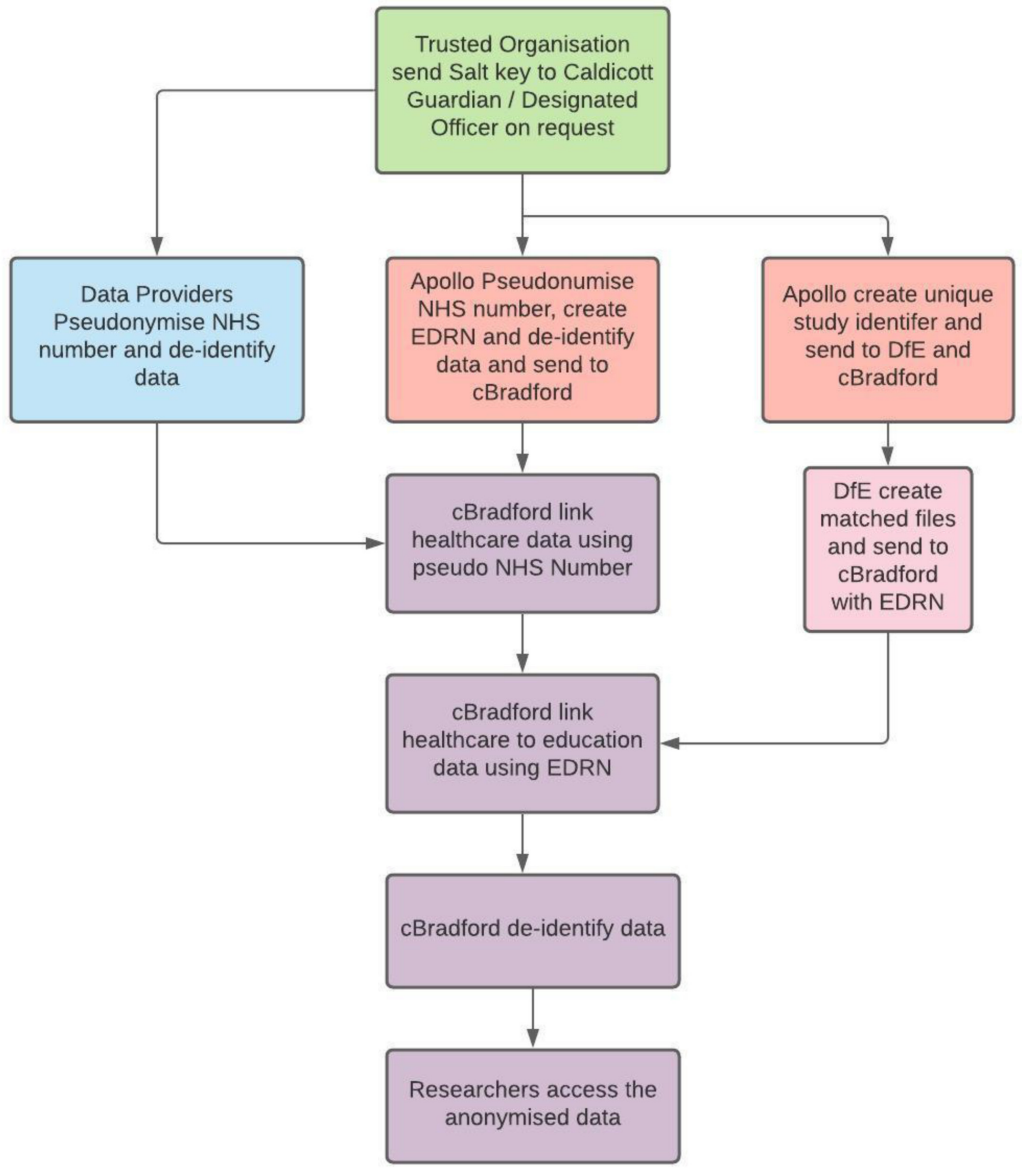
Pseudonymisation process. NHS, National Health Service; EDRN, Education Data Research Number; DfE, Department for Education;

The data providers extracted and pseudonymised the data at source and using secure methods transferred the pseudonymised data to BTHFT where it is linked using the pseudonymised NHS number. In the case of General Practices, the data processor was Apollo Medical Software Solutions Ltd (Apollo). Apollo extracts the necessary pseudonymised primary care information on an automated basis thus providing a fully managed service. An audit trail enabled general practices to see what data and reports are produced and where they have been transferred. The automated process refreshes the cBradford databases on a monthly basis but has the potential to move to a daily basis thereby realising near to real time activity ( Figure 1).

Linkage to non-health sources such as education, employment, benefits data recorded by the local authority, crime and housing, requires matching of non-unique personal identifiers as the unique NHS number is not routinely recorded in non-health datasets. Whilst linkage to aggregate non-health data is supported by matching geographic units, for example using the Lower Super Output Area (LSOA), additional legal bases is required to permit individual data linkage. Education records reference a Unique Pupil Number that identifies each pupil in England and is not available to health care organisations thereby requiring non-unique personal identifiers for data linkage. Confidentiality Advisory Group approval was obtained to permit individual data linkage of cBradford health records to NPD education records and children’s social care data held by the Department for Education using non-unique personal identifiers (CAG ref: 18/CAG/0091 and REC ref: 18/YH/0200).

Future plans for the Connected Bradford Whole System Data Linkage Accelerator include implementation of a process to match individual records to a pseudonymised Unique Property Reference Number (UPRN) to allow individual-level linkage of other non-health datasets such as crime and benefits.

## Data collected

As well as a data provider, BTHFT acts as the controller of the data which is held in a secure environment that adheres to the ISO 27001 standard and to the NHS Data Security and Protection Toolkit ^13^. The database is overseen by the Connected Bradford Research Database Committee (Committee) that provides scientific advice, monitors the secondary use of data for research and service improvement purposes and oversees database development. The Committee includes commissioners, data providers, data governance experts and citizens

**Figure 2.**
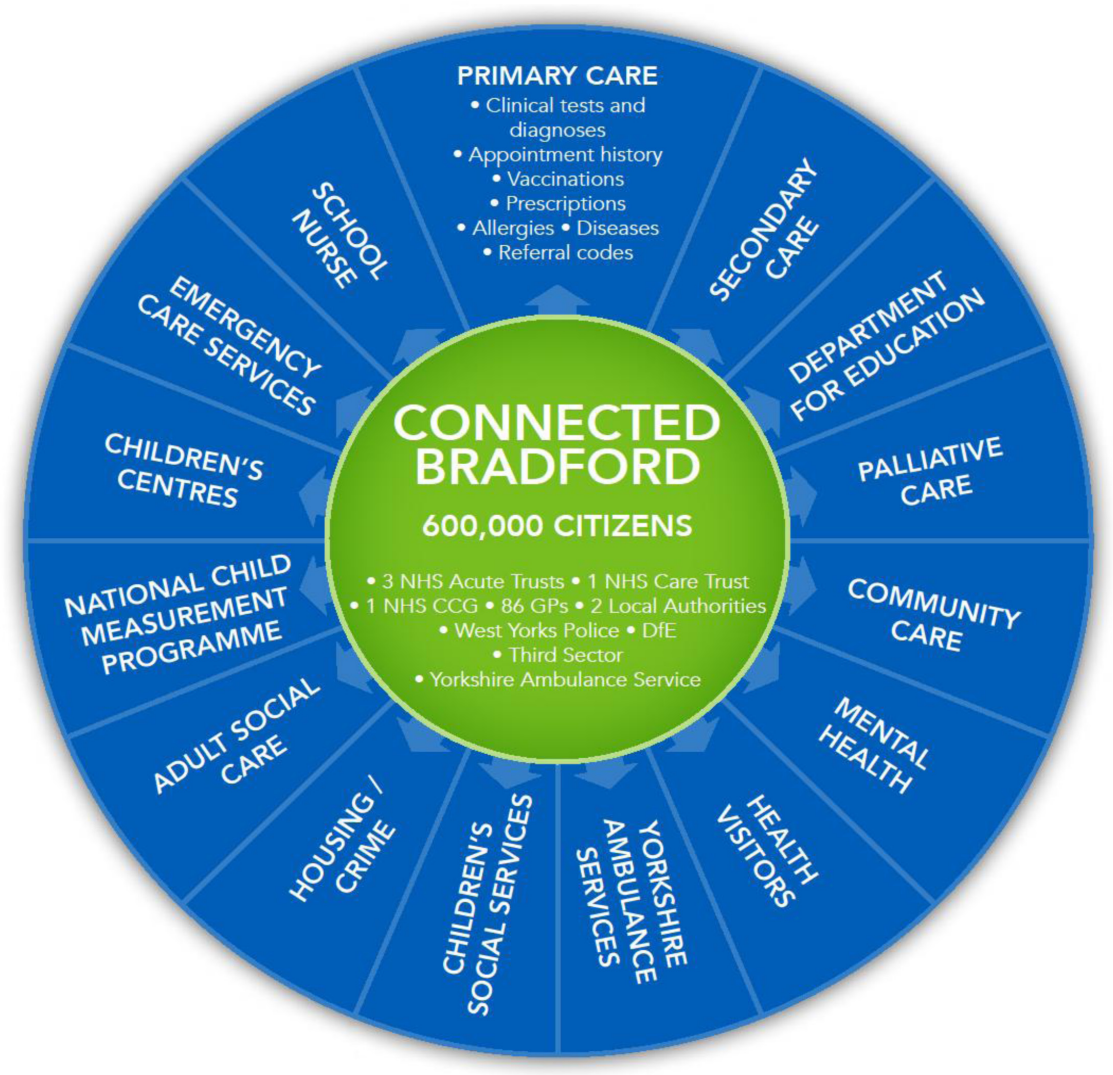
Connected Bradford. NHS, National Health Service; CCG, Clinical Commissioning Group.

The cBradford data linkage model combines primary care data from general practices (including appointment history, prescribing and clinical data), community care data (including mental health, school nurse and health visitor interactions), secondary care data from acute hospitals (including maternity, inpatient, outpatient and emergency services), Yorkshire Ambulance Service 999, Electronic Patient Records, patient transport service and 111 data, palliative care data, adult social care data, children’s social care data, children’s centres data, education, housing and benefits data from local authorities, crime data from West Yorkshire police, housing data from private housing providers and the National Child Measurement Programme data ( Figure 2).

### Patient and public involvement

The cBradford communication strategy focused on improving communication with the local population on the use of health data for research and service development. Existing patient and public panels co-produced posters and leaflets and provided insights on the acceptability of secondary use analysis of data for research and service improvement purposes. A new active cBradford patient and public panel was set up to bring together a network of people to share knowledge and information, co-develop research studies, support interpretation of anonymised health data and discuss future collaborations.

### Key collaborative relationships

To develop data requirements and supporting processes, cBradford has fostered key collaborations across a diverse multi-disciplinary stakeholder group that have been instrumental in the development of the Connected Bradford Whole System Data Linkage Accelerator. Across Bradford, key partners include the Local Medical Committee, Public Health, NHS Bradford District and Craven CCG, Bradford Care Alliance as well as the data providers; BTHFT, Bradford District Care Trust, Airedale NHS Foundation Trust, 86 General Practices, Bradford Metropolitan District Council, Incommunities Ltd (social housing provider) and West Yorkshire Police. Outside of Bradford, we have engaged with Yorkshire Ambulance Service, Calderdale and Huddersfield NHS Foundation Trust, Department for Education who have contributed pseudonymised data towards the Whole System Data Linkage Accelerator as well as Humber NHS Foundation Trust who support the development of the Yorkshire and Humber Care Record ^10^.

This collaborative enterprise has been instrumental in the resulting signed data sharing agreements and automation of data flows by providing exposure to knowledge, expertise, resource investment, opportunities and ways of working between different partners and stakeholders.

## Dataset content and validity

The scale and content of the data are described in Table 1.

**Table 1.** Data Sources, dates, sample size, key variables.

Data Source	Dates	Sample Size	Key Variables	Linkages	Comments
Primary Care	1950– 2021	868,000	Appointments, Medication, Clinical history, Reviews data	Pseudonymised data linkage	GP data for 86 practices across Bradford and Airedale.
Secondary Care	2007– 2021	392,000	Inpatient, Outpatients, Pharmacy, Theatre, Urgent & Emergency Care data	Pseudonymised data linkage	Hospital data from Bradford Hospitals, Airedale Hospital and Calderdale & Huddersfield NHS Foundation Trust.
Community Care	1997– 2020	260,000	Health Visitor, School Nursing, Child and Mental Health Services, Child Development Unit data	Pseudonymised data linkage	Community care data from Bradford District Care Trust.
Adult Social Care	2016– 2020	47,360	Nursing home, Residential home, Homecare data	Pseudonymised data linkage	Adult Social Care data from BMDC and North Yorkshire County Council.
Children Social Care	2005– 2019	315,693	Children in Need, Children Looked After data	Pseudonymised data linkage	Children social care data recorded by the DfE.
DfE education data	1990– 2019	315,693	Early Years Foundation Stage Profile data, Phonics, Key Stage 1,2,4 and 5 data, Absences and Exclusions data	Pseudonymised data linkage	Education data recorded by the DfE.
BMDC education data		30,000	Early Years Foundation Stage Profile data, Phonics, Key Stage 1,2 and 4 data, Absences and Exclusions data	Pseudonymised data linkage	Education data recorded by BMDC.
Yorkshire Ambulance Service	2019– 2021	868,000	Urgent and Emergency Care, 999, 111 data	Pseudonymised data linkage	Urgent and emergency care data recorded by Yorkshire Ambulance Service.
Housing	2020	22,701	Insulation, Type, Rent, Tenancy data	Anonymised	Housing data recorded by BMDC and Incommunities Housing Provider.
Benefits		52,897	Households with income <£15k and >£15k data	Anonymised	Benefits data recorded by BMDC.
Crime	2017– 2020	737,376	Offences, Antisocial behaviour, drugs and alcohol	Anonymised	Crime data recorded by West Yorkshire Police.
Environmental	2020	City-wide	Walkability, Connectivity, Air Pollution, Geospatial coordinates based on UPRN		Environmental data linkages are in progress.

GP, General Practitioner; NHS, National Health Service; BMDC, Bradford Metropolitan District Council; DfE, Department for Education.

Certain threats to validity arise from the nature of routinely collected healthcare data: variation in code selection, the presence of implausible values, missing data, paper record migration and population stability. Practitioner code selection can be affected by factors such as financial incentives, which distort longitudinal disease prevalence trends, and variation in code selection and coding depth between practitioners. Data entry errors and default values incorrectly reported from non-nullable fields can lead to implausible values such as out of range dates and patient ages. Key missing values such as height, weight, blood pressure and socio-demographics can limit some analyses. Although the longitudinal primary care data spans more than forty years in total, the quality and completeness is substantially lower pre-digitisation due to prior data having been transferred from paper records. As such, most analyses will need to focus on data from the past 10 to 15 years to retain validity. Migration in and out of the city means that the population is not stable, as illustrated by the discrepancy between the 868,000 unique individuals in the GP data, and Bradford population figures produced by the Office for National Statistics in 2017 of 534,300, although the expanding geographic footprint of the data explains some of this ^11^.

## Data availability

BTHFT and cBradford are committed to making data available for research and service improvement. cBradford is an ethically approved research database (REC ref: 17/EM/0254). Applications for data access are reviewed every two months by the Connected Bradford Research Database Committee. Further details about how to apply for access to cBradford data are available at https://www.bradfordresearch.nhs.uk/our-research-teams/connected-bradford/

## Discussion

**Figure 3.**
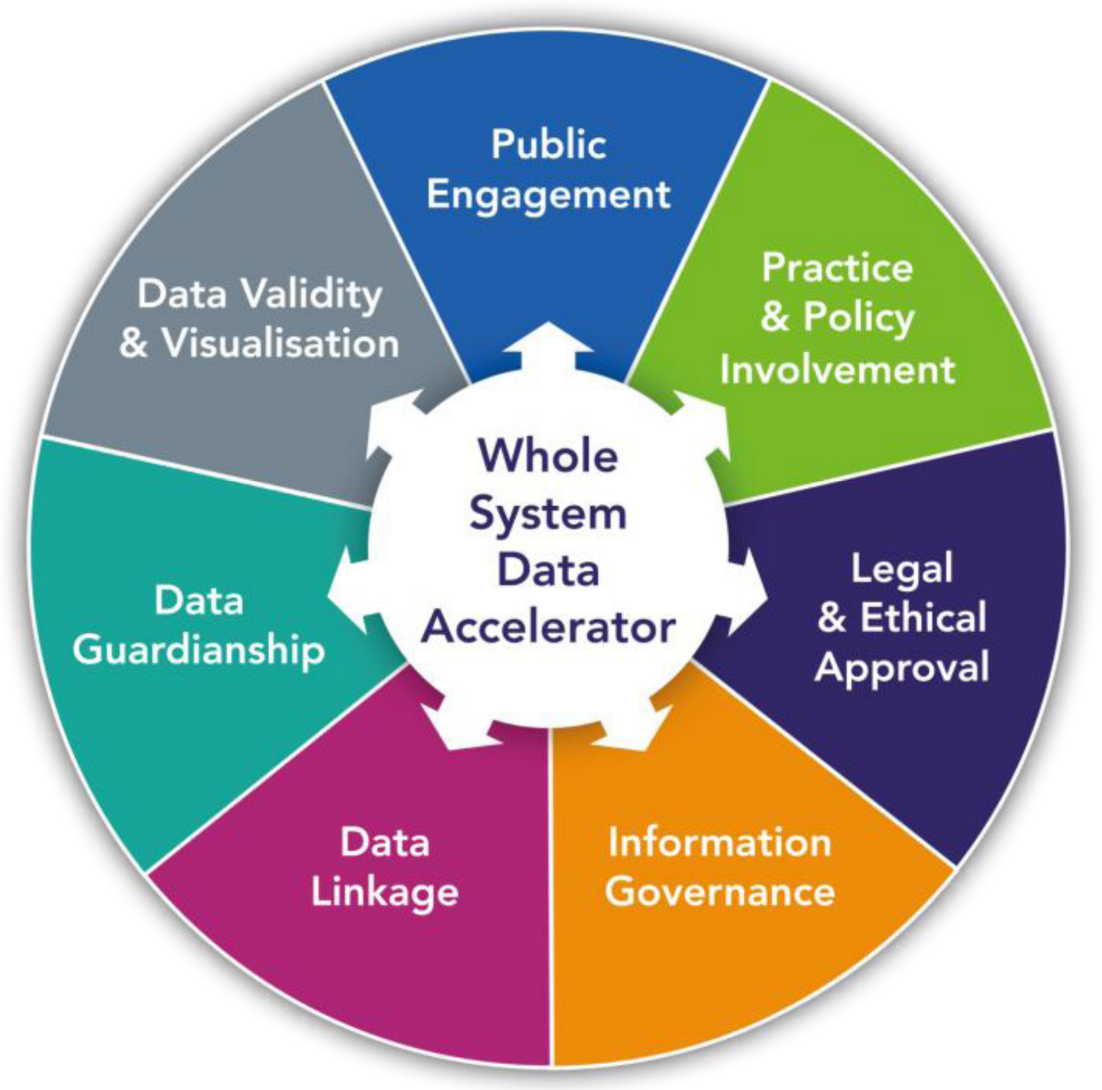
Framework for developing Whole System Data Linkage.

This paper describes the process of linking data from a wider range of health and non-health sources. Connected Bradford has developed from a core of consented Born in Bradford health and education data to cover a whole population system of over 800,000 citizens. There are useful lessons for other districts or cities attempting to establish system-wide data linkage with a number of core themes that we have found to be essential to success ( Figure 3). These are aligned with principles such as FAIR ^14^ and the “five safes,” ^15^ and with the findings of public perspectives on the use of patient data and data-driven technologies using patient data.

**1) Public engagement.** Locality-based approaches to routine data linkage have the advantage of being able to recognise the importance of place and communities. Consultation at a local level on the priorities and objectives, participation in data access committees and open communication of how the data is used to improve services are cornerstones in establishing an acceptable, information governance compliant whole system data linkage. A key challenge is how to ensure sustainable and genuine public engagement that is essential to foster trust in how data is used.

**2) Practitioner and policy integration.** Data providing organisations must invest time and expertise to setup the data extraction process and help interpret the data; therefore it is important that they see some return on that investment. There is a tendency in public sector organisations to use data purely for performance management rather than for improvement. Engagement with health and local government leaders and practitioners across all sectors is essential in identifying how the linked data can support their priorities and objectives, ensuring that analytical work feeds into quality improvement and learning systems decision and supports the best use of limited resources.

**3) Legal and ethical data access frameworks.** Information on the Research Database NHS REC and CAG section 251 approvals is described earlier. The Research Database REC approval ensures that data is available for use by academics and researchers as well as providing reassurance for data providers.

**4) Information governance.** Legal bases, data sharing and processing agreements and the associated transparency and requirements of the Data Protection Act 2018 ^16^, General Data Protection Regulation ^17^ and Common Law Duty of Confidentiality ^18^ represent a potentially significant obstacle to wider access to health, council and social care datasets. With expertise from IG advisors and the Information Commissioners Office we have developed and implemented model Data Protection Impact Assessments (DPIA) and Data Sharing Agreements (DSA) referencing the legal bases and the data linkage process for system partners. One unintended consequence of the Covid19 pandemic has been the greater recognition and support for data sharing.

**5) Technicalities of data linkage.** Unique NHS numbers allow deterministic data linkage for NHS records with NHS numbers encrypted at source with the encryption or salt key held by trusted third parties with Caldicott Guardian approval. These methods ensure high quality linkage and reporting ^19^. Linkage of health to council datasets that do not have an NHS number requires similar approaches to be developed based on address and other patient identifier matching to safeguard confidentiality but has the potential for greater challenges with missing and incomplete data sets.

**6) Data curation, guardianship and access.** Data access for defined purposes is approved by the access committee and enabled using role-based authentication within virtual private networks connections to accredited data safe havens or the trusted research environment. These environments require appropriate analytical tools (e.g. R Studio, STATA) and resource to cover cloud storage. The wide array of databases requires careful curating to promote access and understanding, with clear descriptions, data dictionaries and glossary of codes.

**Figure 4.**
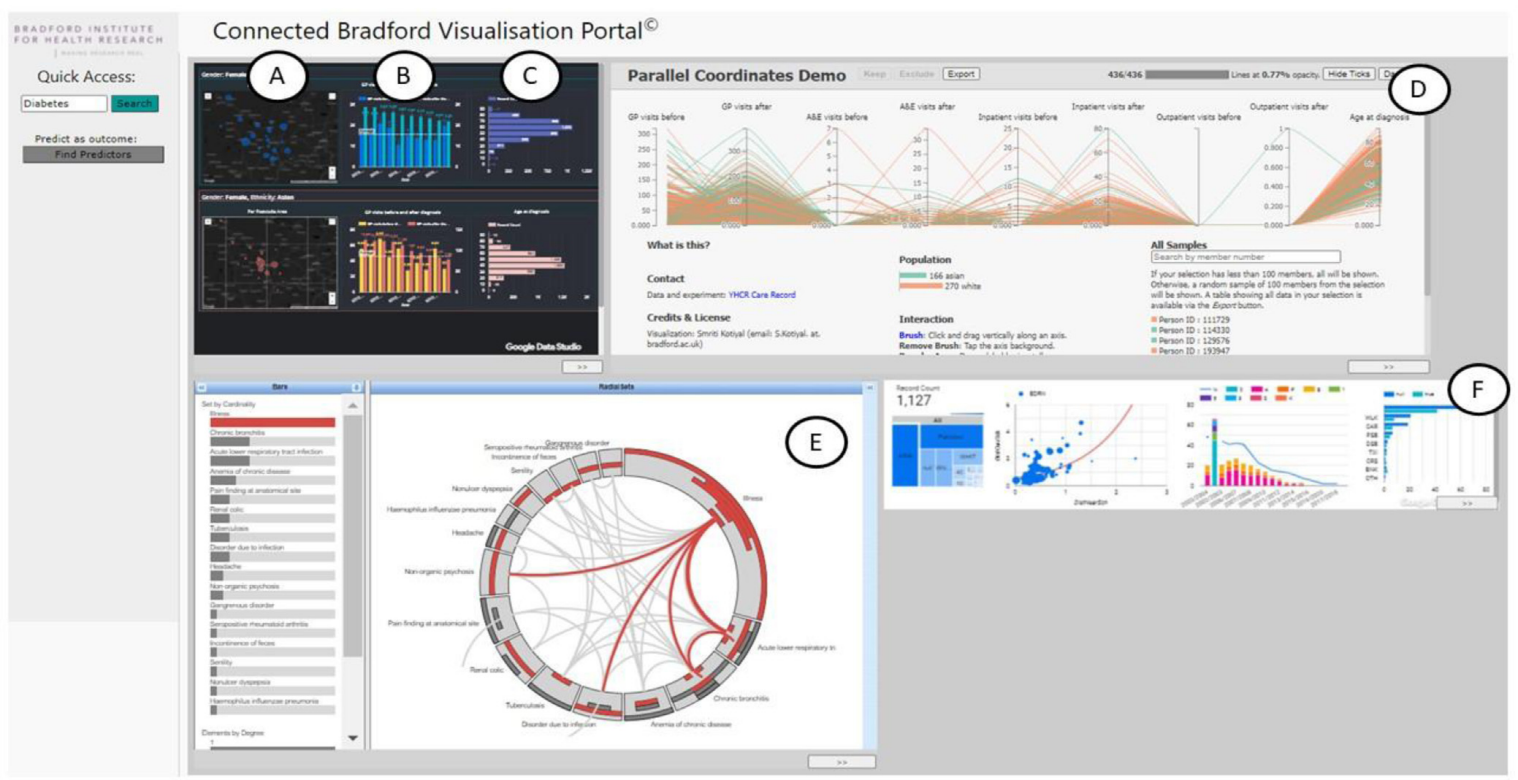
An example workflow connecting Primary Care, Secondary Care and Education data to investigate service demand for women with Type 2 Diabetes. Several visualisation cards have been created in this workflow, starting with a dashboard (top left) showing: ( **A**) the incidence of Type 2 Diabetes for women of White British ethnicity (top row) and Asian / Pakistani ethnicity (bottom row), ( **B**) The bar charts show the number of GP visits before and after being diagnosed broken down by lower-layer super output area (LSOA), and ( **C**) the age distribution at time of diagnosis. ( **D**) A selection of a postcode area (BD4) from the previous card creates a parallel coordinates plot showing the pathways of patients across different services (including GP visits, A&E visits, inpatient and outpatient visits) before and after being diagnosed. ( **E**) Selecting women whose number of GP visits have reduced post diagnosis and who have never been to Accident and Emergency (A&E) before diagnosis creates a new card with a RadialSets visualisation ^1^ displaying the overlap of reasons why those women visited A&E. ( **F**) A small dashboard is created showing education-related measures for people who suffer from headaches, including ethnic background, commute to school, special education needs and their eligibility for free school meals.

**7) Data validity and visualisation.** Key threats to data validity include variation in code selection, implausible values, missing data and population mobility and estimation. A wide range of existing open source and commercial tools and techniques exist to enable opportunities for effective data visualisation, a crucial tool for providing actionable responsive insights for local citizens, practitioners, commissioners and policy makers ( Figure 4). Ongoing involvement of data-providing organisations is a key requirement rather than just the provision of data, as local intelligence on changes to practice or data collection are vital in the context of routinely-collected data.

## Next steps

This paper describes the initial phase of building a whole system linked dataset that we hope will be a useful model for other settings. This is work in motion that will require perseverance and commitment from all the partner organisations and communities if it is to succeed. International technology businesses have gained fabulous wealth from harnessing our private data and the challenge is for public sector organisations to demonstrate how their unique datasets for public good rather than private profit. One of the next phases is to demonstrate the utility and added value of how linked data can improve health and wellbeing through improved understanding of needs and better targeting of support. Our goal is to provide impact studies to illustrate this and demonstrate to local communities how their collective data can provide build a learning health system.

We will also explore the potential to add new layers of data from other partners and from environmental data sources. This could include air pollution, green space, built environment indicators, food outlets and transport to allow us to capture wider influences on health. Finally we are committed to finding better methods to engage our local communities in the nature and benefits of their own data, such as through data visualization and immersive technologies.

## Data availability

This open letter does not include any data. It describes a framework for connecting local data, further details about how to apply for access to these cBradford data are available at https://www.bradfordresearch.nhs.uk/our-research-teams/connected-bradford/
