# Supplementary material for: Connected Bradford:  a Whole System Data Linkage Accelerator
Source: Wellcome Open Res. 2022 Nov 7;7:26. Originally published 2022 Jan 27. [Version 2] doi: 10.12688/wellcomeopenres.17526.2 (PMC9682213; doi:10.12688/wellcomeopenres.17526.2)
Supplement: Peer review history

# Peer review: Reviewer response for version 2

**DOI:** 10.21956/wellcomeopenres.20258.r53194

**Published:** 2022-11-22

**Authors:** Jiang Bian

**Affiliations:** 1 Department of Health Outcomes & Policy, University of Florida, Gainesville, FL, USA

The authors have revised the manuscript and addressed my concerns.

Does the article adequately reference differing views and opinions?

Yes

Are all factual statements correct, and are statements and arguments made adequately supported by citations?

Yes

Is the Open Letter written in accessible language?

Yes

Where applicable, are recommendations and next steps explained clearly for others to follow?

Partly

Is the rationale for the Open Letter provided in sufficient detail?

Yes

Reviewer Expertise:

informatics; ehr; real-world data; data science; AI

I confirm that I have read this submission and believe that I have an appropriate level of expertise to confirm that it is of an acceptable scientific standard.

# Peer review: Reviewer response for version 2

**DOI:** 10.21956/wellcomeopenres.20258.r53195

**Published:** 2022-11-16

**Authors:** Iain E. Buchan

**Affiliations:** 1 Chair in Public Health and Clinical Informatics, Department of Public Health, Policy & Systems, Institute of Population Health, University of Liverpool, Liverpool, UK

The responses are fine.

Does the article adequately reference differing views and opinions?

Yes

Are all factual statements correct, and are statements and arguments made adequately supported by citations?

Yes

Is the Open Letter written in accessible language?

Yes

Where applicable, are recommendations and next steps explained clearly for others to follow?

Partly

Is the rationale for the Open Letter provided in sufficient detail?

Yes

Reviewer Expertise:

public health; health informatics; epidemiology; health services research

I confirm that I have read this submission and believe that I have an appropriate level of expertise to confirm that it is of an acceptable scientific standard.

# Peer review: Reviewer response for version 1

**DOI:** 10.21956/wellcomeopenres.19378.r48337

**Published:** 2022-05-03

**Authors:** Iain E. Buchan

**Affiliations:** 1 Chair in Public Health and Clinical Informatics, Department of Public Health, Policy & Systems, Institute of Population Health, University of Liverpool, Liverpool, UK

The letter describes a population-wide data-linkage platform for healthcare, social care, and other civic functions such as education to combine their intelligence efforts, including inputs from university-based researchers, and co-learn as a civic system.

 An impressive array of data sources is assembled around research (e.g. a birth cohort) and service-development (e.g. educational adjustments) use-cases. The nature of the underlying data construct as a persistent longitudinal multi-agency, multi-purpose civic record and intelligence system could be made more explicit for the international reader unfamiliar with the UK setting. International comparisons might include the PCORnet in the US ( https://pcornet.org/data/) and the Danish national wide-ranging register(s): https://sundhedsdatastyrelsen.dk/da/english/health_data_and_registers.

 The data-linkage, information governance and analysis (pooled and federated) arrangements required to initiate the ‘flywheel’ of a ‘learning system’ are described in general terms. An international comparison might bring out the key component required to pull through timely, frequent analyses (and up-stream data linkage and curation improvements) from service development actions – namely the coupling of population health management with care/service workflow (e.g. https://www.cipha.nhs.uk). The governance required to achieve this must cover analytics sufficiently to manage their quality and trust the results to steer services. The distinction between loosely coupled local policymaking and tightly coupled tactical intelligence might be expanded upon.

 The authors usefully describe the non-unique identifier challenges of linking education and health data. For the international reader unfamiliar with the use of NHS Number as a globally unique person identifier in social care records from non-NHS providers, the authors might explain how service-users are identified, and the consequences of not identifying them properly.

 The authors usefully draw out the distinction between data uses for performance management vs service improvement, and the context of practitioner trust. In addition, the foundation of citizens’ juries in generating the social contract for these data uses is well made. The authors could move beyond public involvement to “foster trust” to active involvement of public members in setting data processing targets such as service improvement intelligence priorities.

 The granularity of analysis-ready data, particularly from secondary care, could be described more for an international audience – detailing the subset of structured, curated hospital episode statistics vs. headroom for more information extraction from clinical narrative etc.

 The geographical granularity of data marked as “anonymised” might be discussed more, for example the small area level at which crime data are available, and their utility in public health intelligence for example.

 The value of the linked data resource is discussed in terms of “public good” or “profit” as a binary distinction, which oversimplifies civic data contexts. For example, the Civic Data Cooperative ( https://civicdatacooperative.com) model may employ social impact enterprise vehicles such as a UK Company Limited by Guarantee specific data uses. The UK civic context of being able to involve data subjects (of records from public services) in closing the ‘data-action gap’ to improve services or enable research through the organised efforts of society is unusual internationally and could be expanded upon.

 This is a big topic that is hard to cover in one letter, the authors raise some important issues and make a valuable contribution.

Does the article adequately reference differing views and opinions?

Yes

Are all factual statements correct, and are statements and arguments made adequately supported by citations?

Yes

Is the Open Letter written in accessible language?

Yes

Where applicable, are recommendations and next steps explained clearly for others to follow?

Partly

Is the rationale for the Open Letter provided in sufficient detail?

Yes

Reviewer Expertise:

public health; health informatics; epidemiology; health services research

I confirm that I have read this submission and believe that I have an appropriate level of expertise to confirm that it is of an acceptable scientific standard, however I have significant reservations, as outlined above.

# Response

**Published:** 2022-08-16

**Authors:** Jane West

**Affiliations:** Born in Bradford, UK

We thank the reviewer for their very helpful comments and have responded below:

1. We are grateful for the suggestion and suggestion and have added this to the introduction.
2. We recognise the importance of valid and reliable data to inform practice and policy. This is core to delivery of any prediction model and ensuring caution and interpretation.
  We have added this to page 2/3.
3. We thank the reviewer for the suggestion and have added this to page 4.
4. This has been added.
5. We agree and have expanded granularity in Table 1.
6. We agree and have expanded granularity in Table 1. We have a data sharing agreement with West Yorkshire Police to provide Connected Bradford anonymised crime data. We are exploring linking this information at a geographical level with other datasets.
7. We have expanded on the Data Action Gap and an amendment has been made in the ‘public engagement’ section.

# Peer review: Reviewer response for version 1

**DOI:** 10.21956/wellcomeopenres.19378.r48336

**Published:** 2022-03-07

**Authors:** Jiang Bian

**Affiliations:** 1 Department of Health Outcomes & Policy, University of Florida, Gainesville, FL, USA

Connected Bradford: a Whole System Data Linkage Accelerator

 This paper describes a large scale effort of linking health care data across different health care systems and then with their non-healthcare data including education, employment, and benefits data, for a large population in Bradford, UK. 

- It is unclear what and how geospatial data are linked to patient data (if by residential history, then need to consider whether the linkage is meaningful; e.g., especially consider that people move around; exposures to poor neighborhood characteristics are only valid, when the neighborhood was poor when the individual was living there).  The temporal component is important for research. 
- Based on the description in the Ethical approval section, it's not clear updates on the database (e.g., adding new borns) need to be re-approved?  (a number of amendments were mentioned for adding additional patients to the database).
- "Unfortunately, there is very little publicly available guidance that sets out the information governance framework to establish a dataset that can be used be for secondary use analysis and research purposes."  Maybe not for this particular context, but information governance does exist (maybe limited), e.g., UK Biobank and various US-based clinical data research networks such as the PCORnet and OHDSI; the All of Us program at US, has pretty complete and well-thought-out data governance guidance. 
- It's not clear what the quality of using the NHS number for linkage; as in the US, the Social Security Number has poor quality (e.g., duplicated assignment exist); and it will be interesting to know (although maybe hard to do) how many data entry errors exist across systems and how will affect the linkage.  At minimum, it's interesting to know whether quality checks exist (e.g., double checking names of those who are linked by NHS numbers). 
- From the description, it seems that individual-level linkage to non-healthcare data does not yet exist, because other data sources do not carry NHS numbers?

Does the article adequately reference differing views and opinions?

Yes

Are all factual statements correct, and are statements and arguments made adequately supported by citations?

Yes

Is the Open Letter written in accessible language?

Yes

Where applicable, are recommendations and next steps explained clearly for others to follow?

Partly

Is the rationale for the Open Letter provided in sufficient detail?

Yes

Reviewer Expertise:

informatics; ehr

I confirm that I have read this submission and believe that I have an appropriate level of expertise to confirm that it is of an acceptable scientific standard, however I have significant reservations, as outlined above.

# Response

**Published:** 2022-08-16

**Authors:** Jane West

**Affiliations:** Born in Bradford, UK

We thank the reviewer for the very helpful comments and have responded below:

1. We are in the process of working with a geospatial data analyst who will be deriving the Unique Property Reference Number (UPRN). The UPRN is a unique number that is allocated to each land or property. The UPRN is routinely recorded by the housing team in the local authority and large private housing associations in Bradford including Incommunities Ltd. In addition to health information, an individual’s address history is available in primary care records. Therefore, we are hoping to have a history of an individual’s pseudonymised UPRN information. The pseudonymised UPRN will be made available to the Connected Bradford programme and will facilitate the data linkage with geospatial data and other data types. This has been added to the ‘Next Steps’ section.
2. We have ethical approval to maintain up to date research databases. Therefore we do not need to seek further approvals to add additional individuals including new-borns to the database. For the following research database, new born babies registered with their general practice or recorded in hospital information will be included. At the moment, the primary care information is received on a monthly basis and the hospital data is received on a daily basis. (IRAS ref:227117 and REC ref:17/EM/0254) The ethics approval  is superseded by the following ethical opinion from August 2022: (IRAS ref:314829 and REC ref:22/EM/0127). For the following research database, we have approval from both the NHS and the DfE to maintain an up to date database which includes all new school entrants. This means that we expect to have the education and health data for all new school entrants children that reach four years old (IRAS ref: 239924, CAG ref: 18/CAG/0091 and REC ref: 18/YH/0200).
3. When we began the development of this dataset in 2016, we identified a number of potential options to develop this database in the UK including an application to NHS Digital for the linked data but this process was potentially lengthy, limited to specific users for specific purposes, costly; this was therefore not going to be sustainable. We also looked for publicly available information on how other organisations or bodies had developed their research databases, but other than information from consented studies such as Born in Bradford, we struggled to get this information. In addition, we noted that the time to develop a large scale consented study was not going to be feasible within the timeframe. We noted that commissioning organisations that had access to linked databases were again limited to whom and for what purpose they could share this data and also had liaised with NHS Digital for this. As a result, we worked with an independent information governance consultant in 2016 who helped navigate the steps, issues, identify mitigations to risks involved, technical processes and other processes involved that arose in the development of the Connected Bradford. These steps have been simplified in the form of the Whole System Data Accelerator framework.
  There has been a drive in the UK over the last few years to record information for individuals using their NHS number. The NHS number is a 10 digit number and there is a degree of validation involved when this information is entered into clinical systems. This has been added to page 4.
4. The Connected Bradford Health and Education Research Database links healthcare data to education data on an individual level. The education data is a non-healthcare dataset. (IRAS ref: 239924, CAG ref: 18/CAG/0091 and REC ref: 18/YH/0200). An individual’s education data does not record NHS numbers and therefore we had to explore an additional technical process which involved additional approvals from the NHS and the Department for Education to support this data linkage. This is described in the third paragraph of the section ‘Pseudonymisation and linkage’

The above responses can also be found in a point-by-point table format here: https://wellcomeopenresearch.s3.eu-west-1.amazonaws.com/linked/269958.Response_to_Reviewer_1_Comments_-Connected_Bradford_0v1_2022-08.docx
